# Insulator Material Deposited with Molybdenum Disulphide Prospective for Sensing Application

**DOI:** 10.3390/mi15121425

**Published:** 2024-11-27

**Authors:** Mariapompea Cutroneo, Petr Malinsky, Josef Novak, Jan Maly, Marcel Stofik, Petr Slepicka, Lorenzo Torrisi

**Affiliations:** 1 Department MIFT, Messina University, V. le F.S. d’Alcontres 31, Agata, 98166 S Messina, Italy; 2 Nuclear Physics Institute of CAS, v.v.i., Husinec-Řež 130, 250 68 Řež, Czech Republicltorrisi@unime.it (L.T.); 3 Department of Physics, Faculty of Science, J.E. Purkinje University, Pasteurova 3632/15, 400 96 Ústí nad Labem, Czech Republic; 4 Centre of Nanomaterials and Biotechnology, Faculty of Science, Jan Evangelista Purkyně University in Ústí nad Labem, 400 96 Ústí nad Labem, Czech Republic; 5 Department of Solid State Engineering, University of Chemistry and Technology Prague, 166 28 Prague, Czech Republic

**Keywords:** pulsed laser deposition, molybdenum disulfide, mass quadrupole spectrometer (MQS), sensor

## Abstract

Two-dimensional molybdenum disulfide (MoS_2_) exhibits interesting properties for applications in micro and nano-electronics. The key point for sensing properties of a device is the quality of the material’s surface. In this study, MoS_2_ layers were deposited on polymers by pulsed laser deposition (PLD). This process was monitored by a mass quadrupole spectrometer to record the emissions of MoS_2_ and evaluate the amount of molybdenum and sulfur compounds generated. The changes in laser parameters during the PLD strongly affect the properties of the formed MoS_2_ film. The exploration of the composition and structure of the films was followed by Attenuated Total Reflectance–Fourier Transform Infrared (ATR-FTIR), Atomic Force Microscopy (AFM), and mass quadrupole spectrometer (MQS). The possible application of the fabricated composite as a sensor is preliminarily considered.

## 1. Introduction

Nowadays, nanomaterials are gaining wide enthusiasm in the scientific community because of their broad applications in sensing, imaging, and synthetic tissue, depending on their engineering [1]. The properties and functionalities of a final product strongly depend on the chemical composition, size, shape, and crystal structure of its constituents. Conventionally, 0D structures, like nanoparticles (NPs), are used in fundamental science [2], nanotechnology, and optics [3] to manufacture active polymers that are able to convert mechanical deformation into electric energy and change the electrical, mechanical, and surface wettability of material. In particular, it has been reported that devices are able to detect external stimuli as a consequence of the variations in the resistances or capacitances of their active surfaces [4,5]. For instance, humidity sensors are based on changes in physical or chemical properties caused by absorbed water molecules [6]. The action operation of the chemo-resistive sensors relies on changes in electrical conductivity/resistivity following the absorption of water molecules on the area of the sensor surface. In fact, the interplay between the adsorbed water molecules and the oxygen induces a change in the charge carrier concentration and then in electrical conductivity/resistivity value [7,8]. Typically, to ensure high performance in humidity sensors, the presence of sensing material with twofold features is required: high surface-to-volume ratio and interior competence. The former is responsible for the effective detection of humidity variation in the environment. The latter interacts with water molecules via physical absorption to assist short response times and the repeated use of the system. To develop sensing materials bearing these features, 2D nanomaterials like graphene have gained extraordinary interest due to their large surface area and unique planar structure. However, the resistance changes in graphene due to humidity alterations are not abrupt, resulting in low sensitivity. Besides the graphene-based material [9], other 2D nanomaterials like the transition metal dichalcogenides (TMDCs) [10] already known since the 1970s [11] with the introduction of the exfoliation techniques for graphene, have been synthesized as monolayers. Owing to their tunable crystal structures and their bandgap depending on their thickness, they re-emerged to fulfill and even exceeded graphene’s expectations. One of the most representative transition metal dichalcogenides, which gathered attention for fundamental study and applications in biomedicine, is MoS_2_. It is a layered material consisting of one layer of transition metal (i.e., molybdenum) sandwiched by two layers of a chalcogenide (i.e., sulfur) by a covalent bond, with a nominal thickness of 0.65 nm and (1.8–1.9) eV direct bandgap [12]. Increasing the number of layers induces a transition from a direct-to-indirect bandgap and a bandgap reduction. Typically, the bandgap is 1.2 eV in the bulk, while the stacked layers are held together by weak van der Waals forces. The comparison between graphene and monolayer molybdenum disulfide (MoS_2_) shows that MoS_2_ is a n-type semiconductor with electron-donating sulfur vacancies. It shows very good mechanical and electrical properties [13,14] and exhibits a significant signal induced by surface-adsorbed water molecules [15,16]. As a consequence of the large surface-to-volume ratio of the MoS_2_ monolayer, its conductivity can be altered by the environment, giving proof of good performance for chemical and biological sensors [12], especially in the presence of intrinsic or extrinsic defects [17]. Choi et al. reported on electrical memory devices providing useful insights into current modulation due to the charge trapping/detrapping density [18]. Ref. [15] describes the film of MoS_2_ quantum dots synthesized in ethanol and water exhibiting a fast response time (11 s), good repeatability, and high stability of sensing even in a low-humidity environment (lower than 37% RH). Late et al. [16] obtained MoS_2_ sheets ranging from single to multiple layers on 300 nm SiO_2_/Si substrates using the micromechanical exfoliation method. They fabricated MoS_2_ transistors assessed for gas-sensing with excellent sensitivity and recovery performances. All these studies stimulated the investigation of MoS_2_ as an active material for sensing applications. This renewed interest came with the demand for optimized production techniques. Alongside techniques such as chemical vapor deposition (CVD), which requires an activation temperature of around 1000 °C, the molecular beam epitaxy (MBE) synthesizes good quality TMDC layers, but with a size smaller than the micron. An effective method enabling the production of high homogeneity growths and quality over micrometric size and high repeatability could be pulsed laser ablation (PLD) [19]. The particles ejected when a laser ablates a solid material reach the substrate and condense on it under a vacuum of the order of ~10^−6^ Torr to produce thin films. The growth of films using PLD typically starts at around 500 °C [20]. The selection of the number of laser pulses can modify the amount of removed material, while the growth speed depends on the pulse rate [21]. The growth of metastable phases and/or crystalline phases, even at room temperature, is assisted by its very high deposition rate and the high energy of ablated material [22]. Moreover, the plasma temperature can be about 100 eV, corresponding to 10^3^ °C, so when the deposition occurs near the plasma, the very high temperature assists the deposition of the crystalline phase. However, the limitation of uncontrollable size and the number of layers on large areas is a huge challenge for MoS_2_ use in a broad range of applications. The current work has been focused on the study of the composition and the quality of the MoS_2_ layers deposited on biocompatible and recyclable insulator material by pulsed laser deposition. The changes in the electronic and chemical properties of the fabricated composite depending on the number of layers produced during the PLD process have been studied for possible sensing applications.

## 2. Material and Methods

The primary target of the molybdenum disulfide (MoS_2_) crystal 99.995% with a size of about 0.5 cm × 1.0 cm × 0.2 mm and a density of 5.06 g/cm^3^ at 15 °C was purchased from Sigma Aldrich. The cyclic olefin copolymer (COC) at 8 μm thick was fabricated as reported in ref. [23]. Polymethylmethacrylate (PET) [24] 10 μm thick was purchased from Sigma Aldrich. The polymeric substrates were cut into pieces of 2 cm × 2 cm.

### 2.1. Pulsed Laser Deposition

The deposition of the adopted insulators occurred in a vacuum of about 7.6 × 10^−3^ mbar inside a vacuum chamber. A Q-switching Nd:YAG laser operating at a wavelength of 1064 nm and a pulse duration of 5 ns was used at a repetition rate of 10 Hz and 30 min of irradiation time. The laser beam passed through a 50 cm lens to focus the beam to a diameter of 0.5 mm on a solid target of MoS_2_ placed in a rotating carrousel to avoid deep ablation, which could modify the laser density. A fresh target surface was always exposed to the incident laser light.

The distance between the window of the incoming laser and the target was 22 cm. The volume of the vacuum chamber was about 52 L. The energy of the laser pulse was 600 mJ, and the laser incidence angle was 45° on the surface of the target, corresponding to a laser fluence of about 76 J/cm^2^. When the laser fluence overcame the ablation threshold of MoS_2_, ions, besides other particles, atoms, and clusters, were ejected. Although controversial results have been published, the experimental results of the author’s research affirm a decrease in FWHM of the angular distribution as the atomic mass of the target and the charge state of the emitted ions increase [25].

In agreement with the literature [26], the ion angular distribution is encompassed by a cosine power law in which the exponent n of the cos^n^ θ distribution gradually increases the atomic mass of elements. In our case, the ion angular distribution in FWHM is assumed to be about ±20° at an on-axis position.

**Figure 1:**
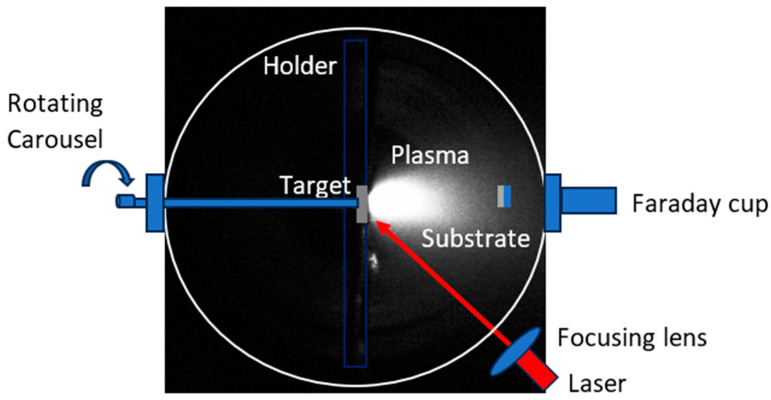
Plasma of MoS_2_ generated by laser during PLD process.

The identification of the laser-generated ions and the evaluation of their energy was performed by an FC located at 45°, as depicted in Figure 1, while the mass quadrupole spectrometer (MQS) and the pump system were located at the top and at the bottom of the vacuum chamber, respectively.

### 2.2. Characterization Methods

To control the quality of the deposition, the generation of ions and neutrals in the plasma source was monitored through the scan of their mass using a mass quadrupole spectrometer. The evolution of the species produced from the plasma laser generated was monitored during single pulses and the repetition rate mode to observe their correlation with the time dependence.

A Tencor P-10 (KLA, Milpitas, CA, USA) was employed as a surface profiler to measure the crater size and shape formed on the surface of the MoS_2_ after one laser shot to evaluate the amount of material removed during the laser irradiation.

A mass quadrupole spectrometer QMG F3 220 PrismaPlus (MQS) from Pfeiffer Vacuum (Aslar, Germany) was used to detect the emitted atoms and molecules from the laser ablation of a target of MoS_2_ in the vacuum. Typically, the spectrometer detects the emission of the gas produced in the vacuum chamber. This spectrometer detects masses ranging between 1 amu and 300 amu, with a sensitivity lower than 1 ppm and a mass resolution better than 1 amu. The MQS is equipped with a SEM detector with a power supply of 1100 V [27]. The masses of interest were selected as follows: 18 (H_2_O), 32 (S), 48 (SO), 64 (S_2_), 96 (Mo), 112 (MoO), 128 (MoS), and 160 (MoS_2_) amu. The MQS detects the emitted atoms and molecules as a function of the time at the laser switching on and the 1 Hz laser repetition rate.

Attenuated Total Reflectance–Fourier Transform Infrared (ATR-FTIR) spectroscopy using a Jasco 4700 and UV-VIS spectroscopy by means of Jasco V750 spectrophotometers (Jasco, Oklahoma City, OK, USA) were employed to analyze the alteration of the presence of specific functional groups, the changes in the chemical structure, and the optical transmittance in the polymer after the deposition of MoS_2_.

The control of the quality of the deposited MoS_2_ was explored by Atomic Force Microscope (AFM) analysis. The accomplishment of the surface average roughness (Ra) and the mean roughness parameter (RMS) was performed using a Dimension ICON-Bruker Corp. (Tucson, AZ, USA). ScanAsyst mode in air was used The 3D images with a scan size of 1 μm were captured using an acquire parameter 1 Hz and processed by NanoScope Analysis software (Tencor P-10).

The monitoring of the electrical capacitance alteration was performed by the I–V characteristic curve through frequency dependence measurements of the output voltage of a high-pass filter at the constant current of 0.5 mA for pristine samples and 0.1 mA for those deposited as described in ref [28]. A metallic mask containing two stripes at a distance of 1 mm from each other and a width of 10 mm was used to assist the production of two gold electrodes, which were 50 nm thick and sputtered on the composite surface. The resistivity of the used resistor was 1 MΩ. The voltage response was recorded over frequencies ranging between 20 kHz and 100 kHz.

**Figure 2:**
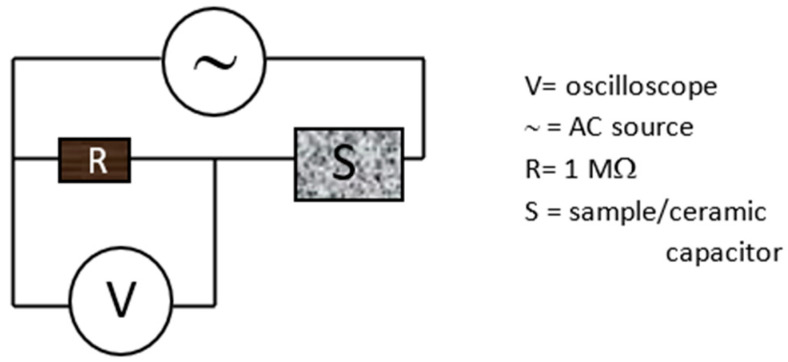
Sketch of the experimental configuration of the electrical characterization of the composites.

The alternating current (AC) with a sinusoidal waveform was generated using a Keithley 6221 current source. The AC voltage vs. frequency was monitored by a Tektronix TDS5104B oscilloscope. Commercial ceramic capacitors of 2.6 pF, 6.8 pF, and 12 pF were used to display the corresponding voltage frequency. In the experimental configuration shown in Figure 2, the prepared composite or the used commercial ceramic capacitor are alternatively positioned.

## 3. Results and Discussion

**Figure 3:**
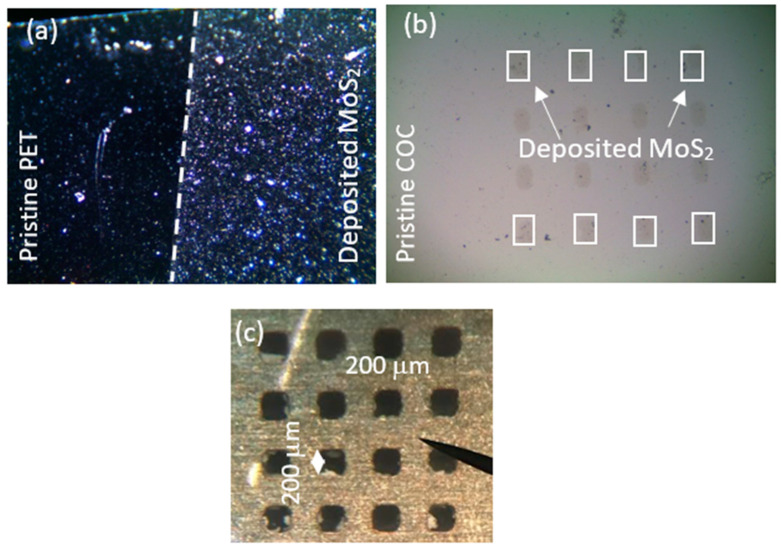
Optical images of PET pristine and deposited with MoS_2_ (**a**), COC pristine and deposited with MoS_2_ (**b**), and the used metallic mask (**c**).

The polymeric substrates of COC and PET were located inside the vacuum chamber both with and without the use of masks and then deposited with MoS_2_ by pulsed laser deposition. Figure 3 reports the optical image 4× magnified of the deposited substrates of PET without the use of a mask (see Figure 3a) and COC covered with a mask (see Figure 3b). Figure 3c shows the detail of a metallic mask, 1 mm thick, at a size of 75 mm × 25 mm, where arrays of squares with a size of 200 μm × 200 μm were machined by laser cutting [29]. In Figure 3a, the areas revealing the pristine PET at the edges of the foil and the region deposited with MoS_2_ were marked by white dashed lines. In Figure 3b, white arrows point out the presence of squared MoS_2_ deposited on COC.

Typically, when a laser irradiates a target and multi-energy and multi-species ions are produced from the laser–matter interactions [30] and deposited on the substrates usually located inside a vacuum chamber. Due to the cruciality of the ion energy in the modification of the structural, physical, and chemical properties of the deposited material, several detectors were employed. Online detectors are the Faraday cup (FC) working in the Time-Of-Flight (TOF) configuration [31] and the mass quadrupole spectrometer (MQS).

**Figure 4:**
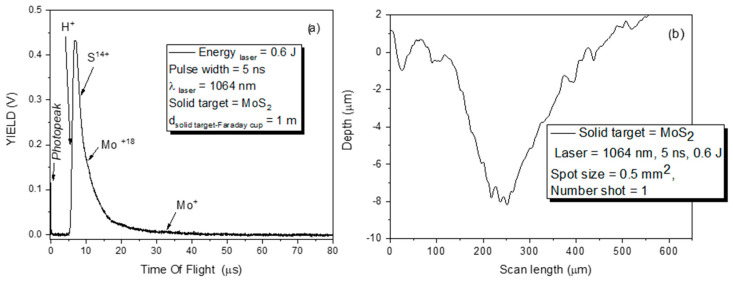
Spectrum of Time-Of-Flight obtained from a Faraday cup when a thick MoS_2_ target was irradiated by a laser (**a**) and the scan of a crater obtained by a profilometer (**b**).

Figure 4a shows the spectrum collected by the FC when the MoS_2_ target was irradiated by a laser beam with an energy of 600 mJ, whereas in Figure 4b, the crater obtained on the surface of the MoS_2_ solid target is shown after one laser shot.

The first peak in the spectrum, which is sharp and narrow and labeled as photopeak, is ascribable to photons produced from plasma and inducing the photoelectron effect, hitting the metallic surface of the FC’s collector. The next structured peak with a long tail is assignable to the detection of protons, sulfur, and molybdenum ions.

This large peak spreading from 5 to about 80 μs is a convolution of signals indicating the presence of protons, sulfur, and molybdenum with different charge states. The maximum energy of the deposited Mo ions is about 3.7 keV, and considering a proton energy of about 200 eV. In agreement with our previous experimental published results [32], it is acceptable to assume that the acceleration of protons and Mo ions is due to the same electric field generated at the surface between the fast electrons emitted and the positive target ions (i.e., sheath surface). This electric field supports an acceleration of about 200 eV/z, where z is the ion charge state. This indicates that the electric field developed at the sheath surface drives the ion acceleration proportionally to its charge state. The results show that Mo ions have a maximum charge state of z = 3.7 keV/200 eV~18.

The energy of Mo, ranging approximately between 205 eV and 3.7 keV, induces a nuclear stopping power higher than the electronic stopping power, which is responsible for the deterioration of the deposited surface, and increased roughness and sputtering effects are expected on the surface of the used substrates.

The maximum energy of Mo ions detected was 3.7 keV, while the maximum energy of the sulfur ions was 2.9 keV. The MoS_2_ was laser-irradiated in a single shot to evaluate the yield of the removed material. Figure 4b shows the crater obtained on the surface of the MoS_2_ primary target at a laser energy of 600 mJ, with a spot diameter of 0.5 mm. From the conical shape of the crater, the estimation of the volume was 5.2 × 10^−7^ cm^3^. Knowing the density of the used MoS_2_, the yield of the removed material per the single pulse seems to be about 2.63 μg.

**Figure 5:**
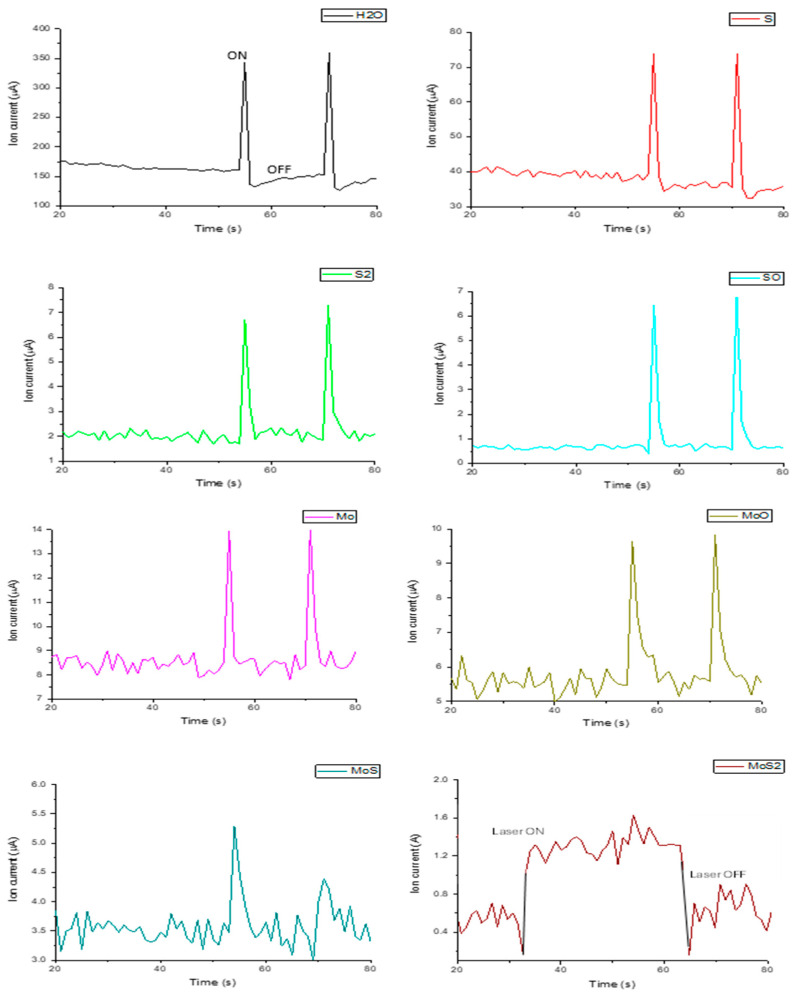
Ion counts vs. atomic mass per unit charge during laser irradiation for repetition rate.

During the PLD processing, the laser irradiation was coupled to a mass quadrupole spectrometer to monitor the ion mass counts in a vacuum. No ions were detected when the primary target was not laser-irradiated, while they were detected in abundance during the laser irradiation, as shown in Figure 5. The spectra of different yields of emitted species are reported as a function of time as the background and yield at the laser switch on and at the laser switch off. The deposition rate of sulfur (S) is one order of magnitude higher than disulfur (S_2_) and sulfur monoxide (SO).

On the other hand, Mo species such as Mo and molybdenum monoxide (MoO) show a deposition rate double that of molybdenum sulfide (MoS), while the MoS_2_ that is slightly detected may be due to the partial sublimation of S, as observed in previous research, according to our published results [33]. The spectra obtained from MQS at the laser switch on and off are summarized in Figure 5. The highest rate of H_2_O displayed in Figure 5 seems to be correlated to the production of ionized hydrogen and oxygen during the laser irradiation of the MoS_2_. The presence of Mo and sulfur, as well as contaminants such as hydrogen and oxygen, is well documented in Figure 5 and is in agreement with the presented FC spectrum (see Figure 4a).

**Figure 6:**
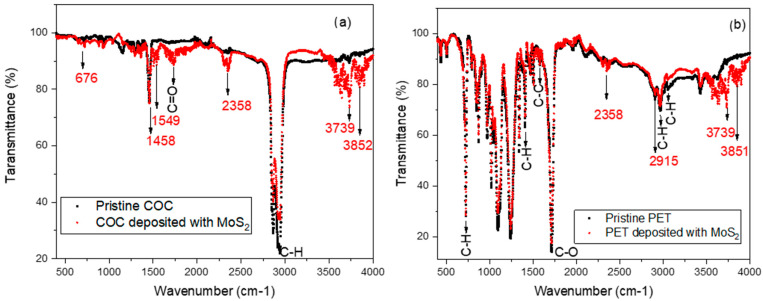
ATR-FTIR spectra for deposited MoS_2_ on commercial COC (**a**) and on PET (**b**).

The molecular structure of the polymeric composites was investigated using ATR-FTIR. It is a powerful method for the effortless identification of polymers of unknown composition and using small samples [34]. The infrared spectrum reported in Figure 6 was carried out on deposited COC and PET foils and compared with their pristine counterparts. Figure 6a shows that the COC has a high transmittance of about 98% in the 4000–400 cm^−1^ wavenumber IR region. The assignments of the stretching vibration bands of the C=O and C-H bonds in the COC [35] segments are at about 1.728 and 2.950 cm^−1^, respectively. The peaks at 3852 cm^−1^ and 3739 cm^−1^ are representative of medium O-H [36] due to the water present in the structure of MoS_2_. The peak at a wavelength of 676 cm^−1^ is due to the Mo-O [37]. The weak peaks at 469 cm^−1^ are assigned to Mo-S vibration [38]. Both in PET + MoS_2_ and COC + MoS_2_, multiple peaks at 700–1150 cm^−1^ can be assigned to sulfate groups [39].

The PET monomer consists of [40] four functional groups: two ester groups, one aromatic ring, and one ethyl group. The two ester groups with the aromatic ring form the terephthalate group, which is linked to the ethyl group to finally form the PET monomer. The functional groups are made of C-C, C-H, C-O, C=O bonds and aromatic rings. Figure 6b shows that the PET has a high transmittance of about 98% in the 4000–400 cm^−1^ wavenumber IR region.

The region from about 1500 cm^−1^ to 500 cm^−1^ is known as the fingerprint region and contains a series of absorptions [41]. The C-O stretching bond of the ester group exists at 1110 cm^−1^ and 1238 cm^−1^. The well-displayed band at 1725 cm^−1^ is due to the stretching vibration of the C=O bond of the ester group. Typically, the absorption bands associated with C=O bond stretching are strong because, in that mode, a large change in the dipole occurs. Concerning the ethyl group, there exists the medium C-H stretching bond at 2970 cm^−1^ and the strong C-H bending bond at 731 cm^−1^. The C-H stretching band of the aromatic ring was found at 3055 cm^−1^. Moreover, the band of the C-H bond stretching vibration of the phenyl ring was found at 1578 cm^−1^, while the band for the C-C phenyl ring stretching band was assigned at 1408 cm^−1^ [42,43].

**Figure 7:**
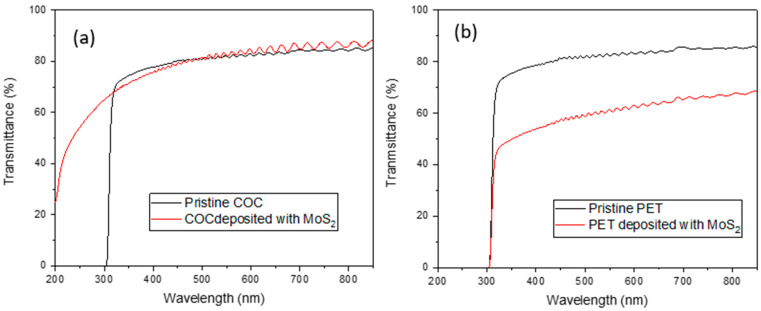
UV-VIS spectra for deposited MoS_2_ on commercial COC (**a**) and on PET (**b**).

In Figure 7a,b, the UV-Vis optical spectra of COC and PET show high transmittance in the region between 350 nm and 850 nm of about 80%. The wavelengths less than 300 nm show high UV absorbance for both fabricated composites.

In the deposited PET (see Figure 7b), a decrease in transmittance is observed with respect to the pristine, while the COC foil deposited with MoS_2_ (see Figure 7a) exhibits higher transmittance than the pristine in the UV region, probably due to the presence of MoS_2_. The COC + MoS_2_ film exhibits minimum surface roughness; thus, it undergoes less incident light scattering and, consequently, higher light transmission [44]. This case improves the performance of electro-optical devices [45]. The transmittance decreases significantly by increasing the thickness of the film. This case is due to stronger incident light absorption in thicker films [46].

**Figure 8:**
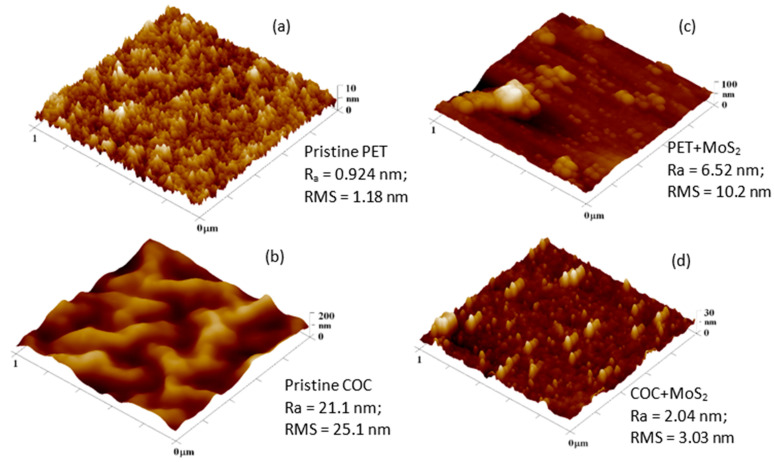
AFM images of PET (**a**) and COC (**b**), for the laser ablated MoS_2_ film deposited onto PET (**c**) and on COC (**d**).

The roughness increases from 0.924 nm in pristine PET to 6.52 nm in the film of MoS_2_ deposited onto PET, as one can see in Figure 8a,c). The mean roughness (RMS) showed an increase of about 88.4% from a value of about 1.18 nm in the PET substrate to about 10.2 nm in the MoS_2_ film deposited onto PET. On the contrary, the roughness decreased to 2.04 nm in the film of MoS_2_ deposited onto COC, as one can see in Figure 8b,d). The mean roughness (RMS) showed a decrease of about 88% from a value of about 25.1 nm in the COC substrate to about 3.03 nm in the MoS_2_ film deposited onto COC. The micrographs show that the film surfaces of COC + MoS_2_, despite the PET + MoS_2_ surface, consisted of grains of almost equal size with a uniform distribution. This discrepancy may be connected to the initial roughness of the pristine PET and COC, providing a different texture assisting the adhesion of the MoS_2_ coating.

**Figure 9:**
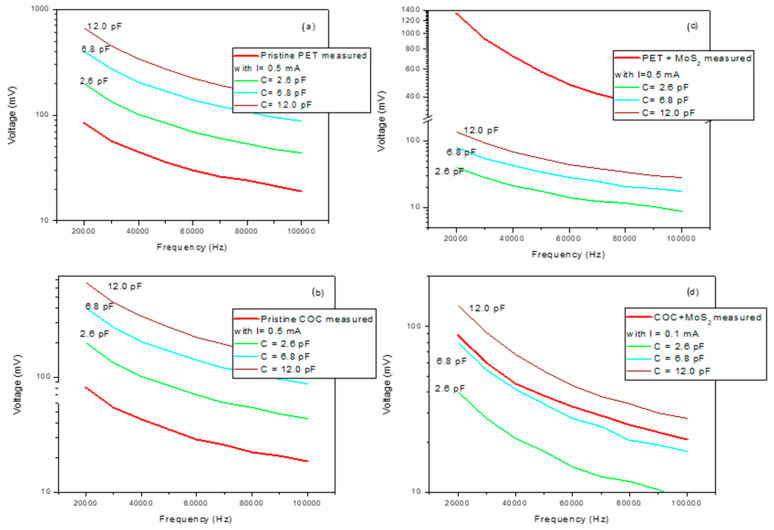
Measures of the electrical capacity on pristine PET (**a**), pristine COC (**b**), PET after the deposition of MoS_2_ (**c**) and COC after the deposition of MoS_2_ (**d**).

The measure of the frequency–voltage response of the COC and PET before and after the pulsed laser deposition process of MoS_2_ was observed and compared to that of commercial ceramic capacitors of 2.6 pF, 6.8 pF, and 12.0 pF, as shown in Figure 9. In Figure 9a,b, an electrical capacity is reported for pristine PET and COC lower than 2.6 pF, which is the minimum value of the commercial capacitors used as a reference. The PET + MoS_2_ exhibits the highest voltage response with a voltage value three times higher than 12.0 pF, while the value evaluated for the COC + MoS_2_ is about 6.8 pF. The trend of the COC + MoS_2_ curve differs from those of commercial capacitors; indeed, it is closer to that of the 6.8 pF capacitor at low frequencies and closer to that of the 12.0 pF capacitor at high frequencies (see Figure 9d). This different slope observed in the curve of COC + MoS_2_ is ascribable to the non-uniformity of the coating. On the other hand, the slope of the PET + MoS_2_ curve is significantly higher than 12 pF (see Figure 9c).

## 4. Conclusions

The composition and quality of the MoS_2_ layers deposited on COC and PET have been analyzed to validate the use of PLD processing for the engineering and characterization of the obtained composites as sensors. PLD is a well-established technique able to control the fast growth of layers of ablated material on all kinds of substrates.

The modification of the MoS_2_ surface and its layer dependence is responsible for a tunable band structure suitable for a broad variety of applications, such as sensors. The performance of the sensor is strongly dependent on the composition of the active area. The analysis of the composition of the deposited MoS_2_ on COC and PET was performed using the FC spectrum and the mass quadrupole spectrometer in vacuum, indicating the formation of sulfur and molybdenum compounds as well as contaminants of hydrogen and oxygen.

Considering the claimed biocompatibility and low toxicity of both COC and MoS_2_, the manufactured composite should be biocompatible too; however, work is in progress to validate these features for promising applications in the biomedical field.

## Data Availability

Data are contained within the article.
